# Exploring the Mumps Virus Glycoproteins: A Review

**DOI:** 10.3390/v14061335

**Published:** 2022-06-18

**Authors:** Jasmine Rae Frost, Saba Shaikh, Alberto Severini

**Affiliations:** 1 Department of Medical Microbiology and Infectious Diseases, Faculty of Health Sciences, University of Manitoba, Winnipeg, MB R3T 2N2, Canada; umfrost3@myumanitoba.ca (J.R.F.); shaikhs@myumanitoba.ca (S.S.); 2 JC Wilt Infectious Diseases Research Centre, NMLB, Public Health Agency of Canada, Winnipeg, MB R3E 3R2, Canada

**Keywords:** mumps virus, glycoproteins, hemagglutinin-neuraminidase protein, fusion protein, epitopes

## Abstract

The resurgence of mumps in vaccinated adult populations has raised concerns about possible waning vaccine immunity or a potential lack of protection to the circulating strain. A number of individual studies have investigated if there are amino acid variations between the circulating wild-type strains and vaccine strains. In these studies, the HN and F mumps surface glycoproteins have been of interest, because of their role in viral infection, and because the HN protein is the target of neutralizing antibodies. Here, we summarize the single nucleotide variants and their potential effect that have been identified between mumps genotypes in the HN and F proteins.

## 1. Introduction

Mumps has seen a re-emergence in recent years, even in highly vaccinated populations. The more recent outbreaks have shown a change in demographic: mumps is no longer a disease of young children, but instead, cases are occurring mainly in young adults [1,2,3,4,5]. What is driving this re-emergence is unknown, but it is likely due to waning vaccine immunity or a lack of cross-reactivity between the circulating strains and the vaccine strain [6,7,8,9].

There are 12 mumps genotypes (A–N), determined by sequencing of the more diverse mumps region, the small hydrophobic (SH) gene [10]. Currently, only six genotypes are circulating world-wide (C, D, F, G, H, and K) [11]. Mumps genotype G accounts for over 50% of genotyped cases world-wide, including those in the outbreaks that have been seen in Canada, USA, the Netherlands and other European countries [12,13,14,15,16]. In Canada and the USA, the mumps component of the MMR vaccine belongs to the Jeryl Lynn strain, which is a mixture of two (JL2 and JL5) closely related genotype A viruses [17]. As the vaccine is a different genotype than the current circulating strain, it is possible that antibodies produced through vaccination are not fully effective against this genotype [6,18]. Current evidence suggests there may be a difference in the ability to neutralize the circulating genotype G compared to the vaccine genotype A. In one study, prior to infection with mumps, individuals had lower antibody titers to a genotype G virus than genotype A [6]. Similarly, it has been shown that there are variances in patient serum in the ability to neutralize a genotype G virus when compared to the vaccine strain [19]. These studies suggest a lack of cross-reactivity, which may be a contributing factor in the more recent mumps outbreaks. Interestingly, there have also been epitope differences identified between the vaccine strain and the circulating strain [8,13,18,20]. These differences could explain the apparent lack of protection from the vaccine.

A similar trend has also been observed in China, where the S79 vaccine (a derivative of the Jeryl Lynn strain) is used. Here, the major circulating strains are of genotype F, but similarly to the outbreaks observed elsewhere with genotype G, differences in neutralization between the vaccine and circulating strain have been observed [21]. One difference between the outbreaks in China and those seen elsewhere is that China currently only has a single MMR dose recommendation in their vaccination plan, which studies have shown is not sufficient to provide full protection. This has allowed mump incidence rates to remain high [21].

The mumps RNA genome is 15,384 nucleotides in length and encodes seven genes: the nucleoprotein (N), the Phosphoproteins (P/V), the matrix protein (M), the fusion protein (F), the small hydrophobic protein (SH), the hemagglutinin-neuraminidase protein (HN) and the large protein (L) [5,22]. The genomic organization of mumps is 3′ N-P-M-F-SH-HN-L 5′, which are on tandemly linked transcription units [22,23]. The two surface glycoproteins of the mumps virus are the HN and F proteins. These glycoproteins are essential for viral entry to host cells, and the spread of newly formed virions [24,25,26].

In addition to being critical for viral entry, the HN protein has been shown to be the target of neutralizing antibodies [27]. These factors, and the fact that a number of B- and T-cell epitope differences have been identified between vaccine strains and circulating strains of mumps, have made these glycoproteins an area of interest in the search to identify what is driving the re-emergence of mumps [13,20,28,29]. The goal of this review is to summarize differences that have been identified in the HN and F mumps proteins.

## 2. Search Strategy

A literature search was performed using the key words: Mumps, MuV, AND Jeryl Lynn, Genotype A, MuVA, Genotype G, MuVG, AND/OR HN, Epitope, Antigenicity, F, Fusion Protein, Hemagglutinin protein, Surface protein, Matrix protein, M protein, Neutral*. The search results are summarized in Table 1. The literature search was performed in September 2020, to OVID MEDLINE^®^ Embase and Scopus. A second search was performed in January 2022 to include more recent literature. Additional articles were selected from the citations of articles from the primary search.

## 3. The Hemagglutinin-Neuraminidase Protein

The hemagglutinin-neuraminidase (HN) protein is a 582-amino acid structural glycoprotein of the mumps virus [30]. HN is a type II membrane protein in which the N terminus is oriented towards the cytoplasm and the C terminus is extracellular [31]. The HN crystal structure has been determined for the genotype B Hoshino strain (Protein Data Bank entry 5B2D and 5B2C) [26]. This structure confirmed that like other paramyxoviruses, the mumps HN head domain shows a six-bladed β-propeller fold (β1−β6 sheets) and forms a homodimer. Two homodimers form a tetramer, again similar to other paramyxoviruses [26,31]. This protein contains a number of conserved motifs: a leucine zipper, the neuraminidase motif and the hemagglutinin receptor binding site (GAEGRV) [32,33].

**Figure 1:**
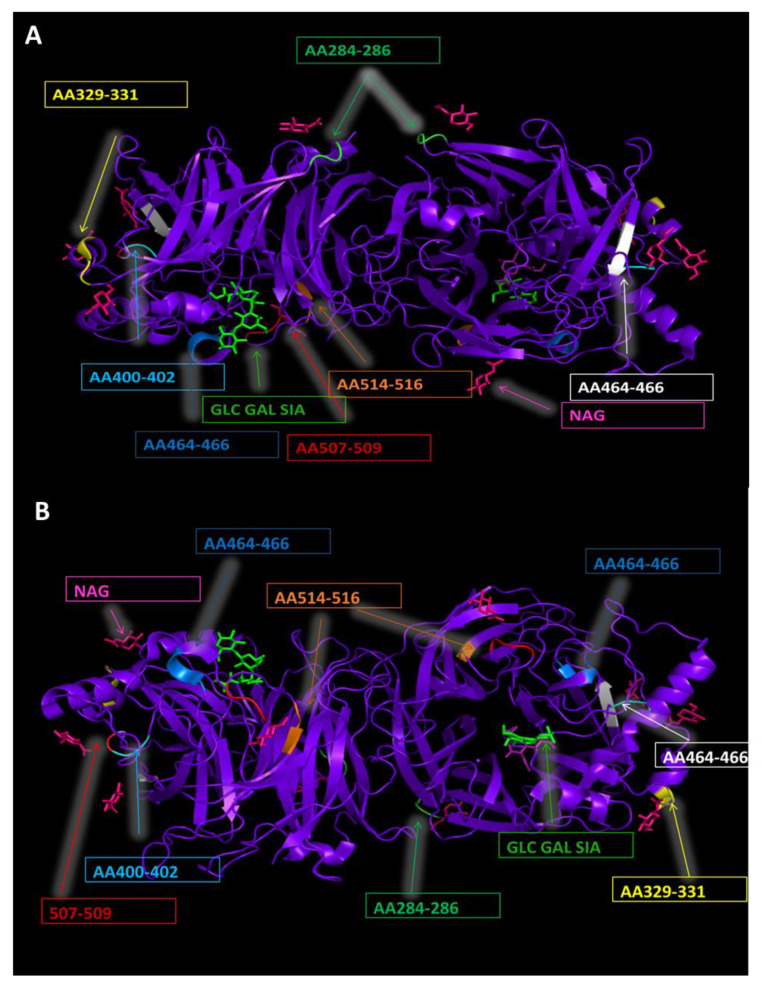
**Protein structures generated from Protein Data Base file 5B2D [26].** (**A**) Protein binding sites and N-glycosylation sites of mumps HN protein (front view). Illustrated are the HN protein (purple), N-acetyl-D-glucosamine binding sites (pink), and sialic acid receptor binding sites (green). Different N-glycosylation sites are labelled with arrows and boxes indicate the amino acid range. (**B**) Protein binding sites and N-glycosylation sites of mumps HN protein (back view). Illustrated are the HN protein (purple), N-acetyl-D-glucosamine binding sites (pink), and sialic acid receptor binding sites (green). Different N-glycosylation sites are labelled with arrows and boxes indicate the amino acid range.

The HN protein exhibits both hemagglutinin and neuraminidase properties and is critical for membrane fusion and viral entry into host cells [34]. The HN protein binds preferentially to trisaccharides with α-2,3 linked sialic acid in unbranched sugars (Figure 1). Binding to the glycan motifs sialyl Lewis^x^ (SLe^x^) and GM2 ganglioside (GM2-glycan) has also been identified [35]. Various sialyated glycan structures can be found in tissues and organs throughout the body, which may explain both the viral tropism for glandular tissues and the spread of mumps throughout the body [26]. The neuraminidase properties of this protein are due to the conserved linear sequence NRKSCS. This sequence is found throughout the paramyxovirus family [32]. The HN protein cleaves sialic acid residues from progeny viruses, preventing agglutination and allowing for viral release from the host cell [26,36]. In coordination with the F protein, the HN protein will mediate both cell to cell fusion and virus to cell fusion allowing for the spread of viral particles to other host cells [5]. The helix-bundled stalk of the HN protein is thought to play a critical role in the activation of the F protein. The dimer–dimer interactions found in the tetrameric head domain of the HN protein seem to be important in the role of the HN protein triggering the F protein for fusion [37,38].

## 4. HN Protein Structure

The HN protein is a major target of immune response in mumps infection [28]. It is also a major target of the anti-mumps antibodies produced by vaccination [39]. It has been shown that there are antigenic differences between HN proteins of different mumps genotypes. In particular, genotype A seems to be antigenically distinct from genotypes B, C and D [40,41,42,43]. A study using monoclonal antibodies raised against the HN protein of a genotype A virus (SBL-1) compared the B-cell epitopes of genotypes A, C, D, G and H viruses and identified different reactivity profiles between the genotype A viruses compared to other genotypes. Additionally, different genotypes have been compared in neutralization tests using rabbit hyper immune sera against genotypes A or D. Here, there was not a clear difference in neutralization patterns between the genotypes C, D, G, H and I. However, genotype A was serologically distinct, showing high neutralization titers against Kilham strain and much lower neutralization titers against SBL-1 or RW (genotype D) strains [40,43].

The mumps HN protein has nine N-linked glycosylation sites at amino acids 12–14, 127–129, 284–286, 329–331, 400–402, 448–450, 464–466, 507–509 and 514–516 (Figure 1) [41,44]. Mutations in sites 12–14 and 127–129, found in genotype G strains, and site 464–466, in vaccine strains (JL5 and JL2), result in loss of N-glycosylation [39,45]. The sites 127–129 and 464–466 can have a cysteine as the variable residues, while site 514–516 can have a proline, which would affect the glycosylation of these sites [41]. The residues 35–53 (ILVLSVQAVILILVIVTLG) are a membrane anchorage site [46]. In a study of the L-Zagreb vaccine strain, it was found that position 51 was an asparagine, not a threonine, while other strains showed this region to be conserved [47]. The effect of this change is unclear.

**Figure 2:**
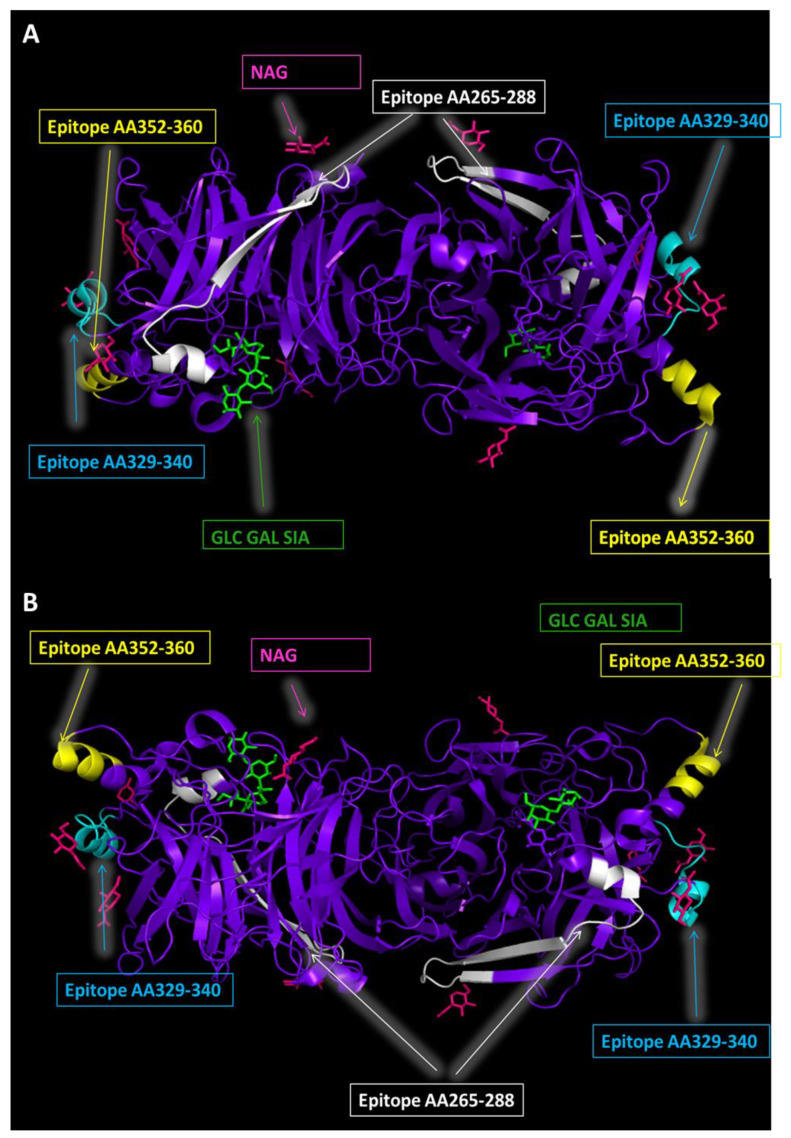
**Protein structures generated from Protein Data Base file 5B2D [26].** (**A**) Protein binding sites and known epitopes of mumps HN protein (Front view). Protein binding sites and N-glycosylation sites of Mumps HN Protein (back view). Illustrated are the HN protein (purple), N-acetyl-D-glucosamine binding sites (pink), and sialic acid receptor binding sites (green). Different epitope locations are labelled with arrows and boxes indicate the amino acid range. (**B**) Protein binding sites and known epitopes of mumps HN protein (back view). Protein binding sites and N-glycosylation sites of mumps HN Protein (back view). Illustrated are the HN protein (purple), N-acetyl-D-glucosamine binding sites (pink), and sialic acid receptor binding sites (green). Different epitope locations are labelled with arrows and boxes indicate the amino acid range.

The regions 265–288, 329–340, and 352–360 on the HN protein have been found to be antigenic (Figure 2) [40,47,48]. When these regions are mapped, they can be seen on the surface of the HN protein, and therefore would be able to interact with antibodies [49]. Within these regions, there are observed amino acid differences between strains (Table 2) [46]. The region between amino acids 329–340 has been used to immunize mice. Antibodies from this immunization were shown to be able to induce neutralizing antibodies against wild-type mumps strains, and thus they could contain a critical epitope for humoral immune response [48]. Single nucleotide variants (SNVs) between genotypes that occur in significant areas of the HN protein have been looked at with interest, as changes in this immune-dominant protein could help explain the re-emergence of mumps that has been seen in vaccinated populations.

## 5. HN B-Cell and T-Cell Epitopes

A number of B- and T-cell epitopes have been identified for the HN protein, either through computational analysis or through in vitro assays. The following regions have been predicted to be B-cell epitopes: 199–207, 220–240 261–266, 269–272, 284–296, 327–331 and 334–363. The regions 261–266 and 269–272 have been experimentally validated to be neutralizing epitopes. This was determined by the production of virus escape mutants, which were then tested for antibody recognition via ELISA using antibodies that had been shown to bind specific regions of the HN protein [40,47,49]. Five T-cell recognition regions have been predicted (74–82, 88–96, 157–165, 326–334, 503–513) of which amino acid variations in four of the regions (74–82, 157–165, 326–334, 503–513) have been shown to reduce HLA binding for genotype G [50]. When the predicted mumps epitopes on the HN protein are mapped, they are predicted to fall near α- helices in the HN head domain. These α- helices show diversity between different genotypes, which may play a role in the outbreaks of mumps that are occurring in vaccinated individuals [26].

The regions 113–130, 375–403 and 440–443 have been identified as potential regions involved in evading neutralization [42]. They have also been described as potential epitopes, and are thought to have a dominant role compared to other epitope sites [42].

A mumps outbreak in Arkansas USA allowed for the opportunity to compare a large number of genotype G strains against the Jeryl Lynn vaccine strain. When B-cell epitopes were predicted for the HN protein, there were clear differences between the circulating genotype G strains and genotype A. A total of 28 amino acid positions were predicted to be in B-cell epitopes on the HN gene. From these, 5 were unique to genotype G strains only (8, 25, 26, 166, and 167), while 17 positions were unique to the vaccine strains, JL5 and JL2 (58, 59, 232, 300, 425, 438, 439, 448, 449, 450, 451, 452, 453, 487, 488, 497, and 532) [13].

## 6. Other HN SNVs

Neuro-virulence had been previously associated with the amino acids 335, 354, 356, 360, 464, and 466 [47,51,52,53,54]. However, conflicting studies have shown that identical mumps sequences can be isolated from patients both with and without neurological symptoms [44]. It seems unlikely these residues alone are responsible for causing the neurological symptoms that are seen in some cases of mumps infection. The actual cause of this is still unknown.

A number of studies have investigated genomic differences between the locally used vaccine strain and the currently circulating local strains. As genotype G strains account for over 50% of cases genotyped world-wide, many studies focus on the differences between this and the Jeryl Lynn vaccine genotype A strain [11].

In the JL5 component of the Jeryl Lynn vaccine, the amino acid 279 is an isoleucine, but in wild-type viruses, it is a threonine. Similarly, position 287 in JL5 is an isoleucine, but in wild-type viruses, it is a valine. These changes have been predicted to result in differences in T-cell and B-cell epitopes, which results in a mismatch of CD4+ and CD8+ responses in an exposure to wild-type viruses [42]. A large study comparing mainly B- and T-cell epitopes of North American sequences (genotype G) to the major component of the MMR vaccine, JL5, found four consistent amino acid changes, which included the positions 279 and 287. The amino acid changes I279T, and I287V have been predicted to be vaccine escape mutations. These changes in amino acids are hypothesized to result in a loss of a T-helper epitope, preventing the induction of B-cell stimulation [29]. The Arkansas study identified SNVs at amino acid positions: I279T, I287V, L336S, and E356D. When the potential structural changes were investigated, they observed that large amino acids were replaced with smaller ones which did result in structural changes [55]. They also hypothesize that the changes L336S and I287V may affect HN protein and antibody binding as these are located in known B- and T-cell epitopes. In particular, the L336S SNV is predicted to strengthen the HN protein as it allows for the formation of added hydrogen bonds with amino acids [55]. Amino acid variations of JL5 compared to other mumps genotypes showed mutations at positions: K74M, R76K, E77A, A80T, A158A/F, H161R, V334I/L, T511A and T513A/I, resulting in reduce HLA binding which could affect T-cell Immunogenicity [50].

A study from the Netherlands comparing circulating genotype G sequences against the vaccine genotype A strain identified eight positions that contained differences, which were located in five known B-cell epitope regions. The amino acid variants found in positions A37V, G63S, H94Y, T97M, T129S, A153S, K317R and S330G were genotype G specific. A number of other variable sites were identified, although not all of them were conserved between all sequences (Table 2) [8].

Additionally, a SNV (N464K) in the HN protein in the Jeryl Lynn vaccine strains JL5 and JL2 can cause a loss in glycosylation. SNVs at position N12S and T129S in genotype G strains have resulted in loss of N-glycosylation. This sites are bordered by neutralizing epitopes [39,47].

Similar to the genotype G outbreaks of mumps seen worldwide, China has experience mumps outbreaks, but of a circulating genotype F virus [56]. In a study of genotype F mumps viruses circulating in China, it was determined that the HN amino acid variants L6F, D25N, V81M, V218A, V249I, T288I, A406S, and T474A increased in frequency in wild-type genotype F sequences from 2001–2015. From this study, it was found that 30 HN SNVs occurred in at least 50% of wild-type samples studied [21]. A second study of strains circulating in China identified the SNV A474V [56].

In a study of HN sequences of mumps viruses found to be circulating in Korea from 1998–2016, (genotypes F, H and I) against the Jeryl Lynn vaccine strain, the authors found no changes in protein glycosylation sites. They did identify the following amino acid variants between the vaccine genotypes and circulating strains: I279T, I287V, L336S, and E356D, in known neutralizing epitope sites. They also identified SNVs in new predicted epitopes/ vaccine escapes sites: N121S, R122K, N123K/E and Y442S [7].

In comparative studies of the Jeryl Lynn vaccine strain against geographic specific circulating strains, the positions 279, 287, 336, and 356 appear often. Table 2 lists all variable sites that have been identified to date [57]. Further investigations are needed to determine if or how these SNVs are playing a role in the mumps outbreaks occurring in young adult populations.

**Table 2:** Currently known HN amino acid variants in wild-type viruses when compared to vaccine strains.

Amino Acid Variant	Position (Amino Acid)	Strain/Genotype	Specific Area in Protein	Potential Effect	Reference
L6F *	6	F	N/A	N/A	[56]
N12S *	12	G	Glycosylation site	Removal of Glycosylation	[57]
D25N *	25	F	N/A	N/A	[56]
A37G	37	G	Membrane Anchorage	N/A	[8]
T51N	51	L-Zagreb	Membrane Anchorage	N/A	[47]
G63S	63	G	N/A	N/A	[8]
K74M	74	I	T-cell Epitope	Change in Epitope	[50]
R76K	76	C	T-cell Epitope	Change in Epitope	[50]
E77A *	77	G	T-cell Epitope	Change in Epitope	[50]
A80T	80	JL2, C, D, F, G, H, I	T-cell Epitope	Change in Epitope	[50]
V81M/T *	81	JL2, C, D, F, G, H, I	T-cell Epitope	N/A	[50,56]
H94Y	94	G	N/A	N/A	[8]
T97M	97	G	N/A	N/A	[8]
N121S *	121	F, H, I	N/A	N/A	[7]
R122K *	122	F, H, I	N/A	N/A	[7]
N123K * N123E *	123	F, H, I	N/A	N/A	[7]
T129S	129	G	Glycosylation Site	Loss of N-glycosylation	[8]
A153S	153	G	N/A	N/A	[8]
S158A/F *	158	K	T-cell Epitope	Change in Epitope	[50]
H161R	161	I	T-cell Epitope	Change in Epitope	[50]
T265A	265	A (SBL-1)	B-cell Epitope	Change in epitope	[49]
V249I *	249	F	N/A	N/A	[56]
I279T *	279	A (JL-5), F	B-cell and T-cell Epitope	Change in epitope (escape mutation)	[57]
I287V *	287	A (JL-2, JL-5, Enders, Rubini, Kilham, SBL-1,), C, D, F, I	B-cell and T-cell Epitope	Change in epitope (escape mutation)	[7,29,55,57,58]
T288I *	288	F	N/A	N/A	[56]
K317R	317	G	N/A	N/A	[8]
S330G	330	G	N/A	N/A	[8]
V334I/L	334	D, H	T-cell Epitope	Change in Epitope	[50]
E335 < K *, E/K335R *	335	B (Urabe), C *, I	B-cell Epitope	Change in Epitope	[10,44]
S336L *	336	A (JL-5), D, F, G, I, J, K	B-cell Epitope	Change in Epitope	[7,11,53,54]
Q354P *, Q354H *, P354H *	354	A (JL2, Enders, Rubini, Kilham *) C	B-cell Epitope	Change in Epitope	[11,44,53,58]
D356E *, E356S *, D356S *	356	A (JL2, JL5, Enders, SBL-1, Rubini, Kilham *) C, F	B-cell Epitope	Change in epitope	[7,11,44,53,58]
A406S *	406	F	N/A	N/A	[56]
Y442S *	442	F, H, I	N/A	N/A	[7]
N464K *	464	A (JL2, JL5)	Glycosylation site	Loss of N-glycosylation	[49]
T474A/V *	474	F	N/A	N/A	[21,56]
T511A	511	J	T-cell Epitope	Change in Epitope	[50]
T513A/I	513	K, F	T-cell Epitope	Change in Epitope	[50]
N523D	523	H (RS-12)	N/A	N/A	[59]

Note: * indicates variant was not conserved in all samples in the study.

## 7. The Fusion Protein

The mumps fusion protein (F) is a 538-amino acid, class one fusion surface glycoprotein [60]. It is responsible for the membrane fusion of virus and host cell [61]. The un-cleaved protein has three hydrophobic regions: an amino-terminal signal peptide, an amino terminal region of F1 and the carboxyl-terminal membrane domain [62]. This protein starts as a precursor molecule (F0), and is then cleaved into the active protein by the recognition of a R-X-L/R-R motif by a host endoprotease (furin) [63]. The F protein contains two disulfide-linked polypeptides (F1 and F2) [61]. The fusion core from the mumps Miyahara strain (genotype B) has been crystalized (protein data base entry 2FYZ) [61]. The structure of the mumps F protein is similar to other paramyxovirus. This glycoprotein has two discontinuous heptad repeat domains at the ectodomain [61,64]. The core complex forms a six-helix bundle, and forms a 3-4-4-4-3 spacing that has also been seen in other viruses such as RSV [61].

The role of the fusion glycoprotein is to mediate the fusion of lipid membranes, and this occurs at a neutral pH. Binding of the HN protein to a receptor signals the F protein to undergo the conformational change needed to drive the fusion of the viral and cell membranes [24,25].

## 8. F Protein Structure

In the mumps fusion protein, the first 19 amino acids act as a signal peptide to promote the F protein function [62,65]. This sequence has been shown to vary between strains, unlike the region 98–102, which is recognized by the cell proteases and is strongly conserved [46]. Residues 483–512 (IGAIICAALCLSILSIIISLLFCCWAYIAT) comprise a membrane anchorage [62]. In the L-Zagreb strain position 489 contains a threonine not an alanine [46].

The amino acid 195 is known to play an important role in the fusogenicity of the virus [66]. In the L-Zagreb and Urabe strains, there is a phenylalanine in this position, while the Jeryl Lynn components, Rubini, Hoshino, and Miyahara strains, have a serine [46].

The fusion protein also contains glycosylation sites at positions: 73–75, 182–184, 352–354, 427–429, 433–435, 457–459 [62].

## 9. F Protein Epitopes

A number of B-cell epitopes have been identified for the mumps F protein. In a study using anti-mumps mAbs, the amino acid positions 221, 323, and 373 were determined to be located in at least two conformational neutralization epitopes. Glycosylation at position 373, or the loss of glycosylation at position 323 in genotype G strains, resulted in a mechanism of escape from the mAbs [67]. Using prediction software to model the F protein, it has been determined that residues 221 and 373 are distant and must be on separate epitopes in the monomeric form. However, in a homotrimer model, these residues can be found in a closer proximity to each other [67].

A comparison between genotype G sequences from a mumps outbreak in Arkansas and the components of the MMR vaccine (JL5 and JL2) found distinct predicted B-cell epitope amino acid positions on the F protein. From the 17 total predicted positions, genotype G predicted B-cell positions were found at amino acid positions: 19, 88, 159, 173, 174, 353, 368, 435, 461 and 515. Positions 173 and 368 were unique to the wild-type genotype G viruses. The vaccine strains also had unique predicted epitope positions at amino acid positions 18, 27, 324, 344, 350, and 412 [13]. These areas will be of interest in future studies, as structural differences in B- and T-cell epitopes could lead to a change in immune system recognition.

## 10. F Protein SNVs

Similar to the mumps HN protein, many studies have explored differences in the Jeryl Lynn vaccine strain to the circulating strain, often genotype G (Table 3). Differences between genotype A mumps and genotypes D, C and B have been noted at amino acid positions T7I, I13V, V49I, S318R, S345T, A409S and N480S. Some genotype C and D samples showed alternate variants at positions S318G and A409T [65]. Interestingly the variants in amino acids T7I and I13V occur in the signal peptide of the N terminus. Similar to what has been observed with the HN protein, it was determined that the mumps A genotype was not as closely related as the B, C and D genotypes [65]. A study comparing circulating genotype G strains against the genotype A Jeryl Lynn vaccine strain in the Netherlands identified the mutation S97L as a SNV of interest. As this variant is found near the cleavage site that results in the conformational change in the F protein, the authors hypothesized it may play a role in enhancing the fusion process because of the increase in hydrophobicity [8].

Other variants of note include the strain RS-12, which has an isoleucine at position 269, instead of the methionine found in circulating sequences [59]. Additionally, a study of circulating genotype F strains in China found the following SNVs: V151I and H329Y [56]. How these amino acid variants are affecting immune responses in individuals is not yet clear.

**Table 3:** List of known F protein amino acid variants and their properties.

Amino Acid Variant	Position (Amino Acid)	Strain/Genotype	Specific Area in Protein	Potential Effect	Reference
T7I	7	A (SBL-1, Enders, Kilham)	Signal peptide region	N/A	[65]
V13I	13	A (SBL-1, Enders, Kilham)	Signal peptide region	N/A	[65]
I49V	49	A (SBL-1, Enders, Kilham)	Signal peptide region	N/A	[65]
S97L *	97	G	Close to cleavage site	N/Aouma	[8]
V151I *	151	F	B-cell Epitope	N/A	[56]
S195F *	195	B (Urabe), C, D, F G, I, N, L-Zagreb	N/A	Increase Neurovirulence and Fusogenicity	[10,24,47,53,65]
M269I	269	H (RS-12)	N/A	N/A	[59]
S318R, S318G *	318	A (SBL-1, Enders, Kilham), C *, D *	Signal peptide region	N/A	[65]
H329Y *	329	F	N/A	N/A	[56]
S345T *	345	A (SBL-1, Enders, Kilham),	Signal peptide region	N/A	[65]
A409S, A409T *	409	A (SBL-1, Enders, Kilham), C *, D *	Signal peptide region	N/A	[65]
N480S *	480	A (SBL-1, Enders, Kilham),	Signal peptide region	N/A	[65]
A489T *	489	L-Zagreb	Membrane anchorage	N/A	[47]

Note: * indicates the mutation was not conserved between samples in the study.

## 11. Discussion

The re-emergence of mumps is a trend that has been seen worldwide, and is occur-ring in vaccinated young adult populations [4,15,16,68,69,70]. These outbreaks are caused by a mumps genotype that differs from the vaccine genotype used in that region. Most outbreaks are due to a mumps genotype G, while the vaccine that is used in the same area is the Jeryl Lynn strain, which is a mixture of two genotype A strains (JL5 and JL2) [11,12,16,71]. The factors that are driving these outbreaks are unclear, but there is evidence to suggest a difference in cross-reactivity or a difference in the viral epitopes may be playing a role [6,19].

Differences in neutralization between vaccine strains and circulating strains have been observed in many studies [8,42,72,73]. The reason for these differences is not well understood, but one popular hypothesis is it could be due to the differences in the functional or immunological properties of the mumps HN and F glycoproteins. A number of difference have been identified in the mumps HN and F glycoproteins. A large study conducted in the USA found 32 amino acid site substitutions between their circulating stains and the Jeryl Lynn HN protein (the component of the vaccine used in the USA). The majority of the amino acid variations were conserved between the outbreak sequences and a previously sequenced strain that had been shown to have a lower degree of neutralization than the Jeryl Lynn strain [8,19,70].

## 12. Conclusions

While it is still unclear if the changes in the mumps glycoproteins are the cause of recent outbreaks, the number of differences observed between vaccine and circulating strains suggests this could be a factor. Future studies will need to be carried out to determine how the changes in glycoproteins effect antibody recognition. Screening for future variants will also be important in subsequent outbreaks. This review summarized what is known to date on the mumps HN and F glycoproteins and their epitopes.

## Figures and Tables

**Table 1:** Search terms and results for the literature search of mumps epitopes.

#	Searches	Results
1	* mumps/or mumps virus/	4617
2	(mumps or parotit *).tw,kw.	10,226
3	or/1–2	10,979
4	exp mumps vaccine/or exp vaccination/or genotype/	553,092
5	“virus neutralization”/ or“neutralizing antibody”/	44,343
6	(Jeryl Lynn or genotyp * or strain * or vaccin * or immuni * or neutral *).tw,kw.	1,998,068
7	or/4–6	2,103,476
8	exp glycoprotein/ or exp fusion protein/ or HN protein/ or exp membrane protein/ or matrix protein/ or virus protein/	1,593,994
9	((f or fusion or HN or hemagglutinin or membrane or surface or m or matix or virus or viral) adj3 (protein? or glycoprotein?)).tw,kw.	312,005
10	(haemagglutinin neuraminidase or haemagglutininneuraminidase or glycoprotein?).tw,kw.	177,388
11	or/8–10	1,810,253
12	exp antigenicity/ or epitope/	150,132
13	(antigenic * or immunogenetic * or immunogenic * or epitop *).tw,kw.	251,994
14	(antigen * adj2 (strength * or activit * or propert * or determinant *)).tw,kw.	19,464
15	or/12–14	297,523
16	and/3,7,11,15	88
17	limit 16 to (english and yr = “2000–Current”)	65

Note: * Represents the truncation symbol used in the databases.

## Data Availability

Not applicable.
